# Evaluation of subconjunctival liposomal steroids for the treatment of experimental uveitis

**DOI:** 10.1038/s41598-018-24545-2

**Published:** 2018-04-26

**Authors:** Chee Wai Wong, Bertrand Czarny, Josbert M. Metselaar, Candice Ho, Si Rui Ng, Amutha Veluchamy Barathi, Gert Storm, Tina T. Wong

**Affiliations:** 1 Singapore National Eye Centre (SNEC), 11 Third Hospital Avenue, Singapore, 168751 Singapore; 2 Singapore Eye Research Institute, 11 Third Hospital Avenue, Singapore, 168751 Singapore; 3 Department Pharmaceutics, Utrecht Institute for Pharmaceutical Sciences (UIPS), Utrecht University, PO Box 80082, 3508 TB Utrecht, The Netherlands; 4 School of Materials Science and Engineering (MSE), Nanyang Technological University, 11 Faculty Avenue, Singapore, 639977 Singapore; 5 Lee Kong Chian school of medicine (LKCmedicine), Nanyang Technological University, 11 Mandalay Road, Singapore, 308232 Singapore; 6 Department of Experimental Molecular Imaging, University Clinic and Helmholtz Institute for Biomedical Engineering, RWTH Aachen University, Aachen, 52074 Germany

## Abstract

Non-infectious anterior uveitis (AU) is a potentially sight threatening inflammatory condition. The current gold standard for treatment is topical steroids, but low ocular bioavailability and compliance issues with the intensive dosing regimen limit the efficacy of this treatment. Liposomes as a drug delivery system may help to overcome these problems. We studied the efficacy of a PEG-liposomal formulation of liposomal steroids, administered as a single subconjunctival dose, in the treatment of experimental uveitis in rabbit eyes. Rabbits that received subconjunctival liposomal triamcinolone acetonide phosphate (LTAP) or liposomal prednisolone phosphate (LPP) had significantly lower mean inflammatory scores than untreated controls on Day 4 after induction of uveitis (LPP vs controls, p = 0.049) and 8 (LPP vs controls, p = 0.007; LTAP vs controls, p = 0.019), and lower scores than rabbits given topical PredForte1% 4 times a day on Day 8 (p = 0.03). After antigen rechallenge, the subconjunctival liposomal steroid groups continued to have greater suppression of inflammation than untreated controls on Day 11 (p = 0.02). Localization of liposomes in inflamed ocular tissue was confirmed by histology and immunostaining, and persisted in the eye for at least one month. Our study demonstrates that a single subconjunctival injection of liposomal steroids induces effective and sustained anti-inflammatory action.

## Introduction

Non-infectious anterior uveitis (AU) represents a group of immune-related, sight-threatening inflammatory conditions that account for 60% of all cases of uveitis seen in eye centres^1–5^. Sight threatening eye complications can occur upon prolonged uncontrolled inflammation, including cataract, glaucoma, and swelling of the central retina. These complications lead to blindness in up to 25% of patients^6,7^. Corticosteroids are the first choice treatment for anterior uveitis, and the current gold standard treatment is topical eyedrops therapy^8^. However, several limitations exist for topical eyedrop administration^9^: 1. Bioavailability is poor due to limited capacity of the conjunctival sac (25 μl), short precorneal drug residence time and drainage of drug via the nasolacrimal duct. It has been estimated that the ocular absorption of topically applied drugs is less than 5%^10^; 2. Steroid eye drops are suspensions, which can cause blurring of vision and ocular irritation^11^; 3. Intensive treatment is required, resulting in a challenge to comply with the treatment regimen. These factors combine to limit the efficacy of topical eyedrop treatment, resulting in persistent inflammation and sight threatening complications related to chronic inflammation. In addition, the untargeted delivery of steroids to uninflamed ocular tissue can result in steroid related side effects such as cataract^11^ and glaucoma^8^.

To avoid the problems of poor bioavailability as well as the side effects, various drug delivery systems have been studied for treating ophthalmic diseases, including polymer- and lipid-based nanomaterials^9,12–14^. The most studied nanocarriers in ophthalmic disease are liposomes, which have the advantages of being biocompatible and biodegradable^14^. Some liposomal formulations are already used in clinical trials for eye diseases^15,16^. Different routes of delivery and formulations have been developed to optimize the delivery of liposomal drugs into the anterior or posterior segment of the eye by altering the surface charge or lipid composition^15,17^. The use of liposomal formulations of vasoactive intestinal peptide^18,19^, dichloromethylene-diphosphonate^20–23^ and FK506^24^ for the treatment of experimental uveitis have been previously reported. Our study is the first to assess the effectiveness of liposomes to deliver a single subconjunctival dose of a well-established treatment (steroid), in comparison to a single injection of unencapsulated steroid and to the current gold standard of intensive topical steroid eyedrops.

In this study, we employed pegylated liposomal formulations of water-soluble corticosteroid derivatives, notably prednisolone phosphate and triamcinolone acetonide phosphate, both active ingredients know to be effective in free form for the treatment of AU in humans. PEGylation of liposomes enhances their bioavailability by increasing solubility, decreasing enzymatic degradation and reducing clearance^25^. Pegylated liposomal formulations of water-soluble corticosteroids have already shown promising results in human trials to treat systemic inflammatory diseases such as rheumatoid arthritis (Phase I/II) and ulcerative colitis (Phase IIa)^26,27^. Interestingly these liposomes have been shown to be efficiently and specifically taken up by macrophages in inflamed tissue^22,28,29^. When injected in the subconjunctival space, we postulate that, besides creating a local depot providing sustained release of the drug, the liposomes could also enhance uptake of the drug by local inflammatory target cells in AU. This is a therapeutic efficacy study with a GMP-liposomal corticosteroid formulation that has shown to be active in a variety of models of chronic inflammation after IV administration, by virtue of targeting to the inflamed target sites^26,27,29–31^. In this study, we compared the efficacy of subconjunctival liposomal prednisolone phosphate (LPP) and liposomal triamcinolone acetonide phosphate (LTAP) with topical prednisolone acetate 1% eyedrops and subconjunctival free prednisolone phosphate for the treatment of AU in a rabbit model of experimental uveitis.

## Materials and Methods

### Animals

Approval was obtained from the SingHealth Institute Animal Care and Use Committee (IACUC Singhealth Approval Number 2016/SHS/1184) and all procedures were performed in accordance with the ARVO Statement for the Use of Animals in Ophthalmic and Vision Research. 26 Adult New Zealand White rabbits, weighing 2–2.5 kg were used in this study. All rabbits were examined with a slit lamp and only rabbits with no ocular pathology were included in the study. Rabbits were randomized into one of the following arms: Subconjunctival liposomal prednisolone phosphate, subconjunctival liposomal triamcinolone acetonide, subconjunctival free prednisolone phosphate, topical prednisolone acetate 1% and no treatment.

### Liposomal steroid preparation

Liposomes were prepared as previously described^30^. In brief, dipalmitoyl phosphatidyl choline (DPPC), cholesterol, and PEG2000 distearoyl phosphatidylethanolamine (PEG-DSPE) were added in a 62%, 33%, and 5% molar ratio. Steroids were dissolved in water for injection while the lipids were dissolved in absolute ethanol at 65 °C. The alcoholic lipid solution was injected in the aqueous steroid solution and mixed under heating to 65 °C, forming a multilamellar vesicle dispersion. This dispersion was downsized to the desired particle size of approximately 100 nm in diameter by repeated homogenization cycles using an Avestin C55 high-shear homogenizer (Avestin, Mannheim, Germany). Unencapsulated steroids were removed by ultrafiltration using membranes with a molecular weight cut off of 30 kDa and replaced with clean dispersion buffer. Finally, the liposomal dispersion was sterile filtered, collected in vials and stored between 2 and 8 °C. Cyanine 5.5 and Fluorescein isothiocyanate (FITC) liposomes were prepared identically with the addition of 0.2% of DSPE-CY5.5 or FITC as described by Lobatto *et al*.^31^. The characteristics (Table 1) and drug release profile of this formulation in aqueous medium and plasma has previously been published. In such media, they show good drug retention properties, which is essential to ensure transport and delivery of the liposomal encapsulated drug at the target cells (e.g. macrophages) in the inflamed site^26,27,29^. The formulation studied here is the same as the formulation developed and evaluated by Lobatto *et al*. With this formulation, neither *in vitro* (buffer, 37 °C) nor *in vivo* (in the blood circulation) release of encapsulated drug from the liposomes was observed^31^. However, despite the complete stability of the liposomes *in vitro* and in the circulation, low levels of free drug were detected in plasma, which are due to liposome clearance from blood and subsequent drug release by liver and spleen macrophages back to the circulation^26^.

**Table 1:** Characteristics of liposomes.

	Empty liposomes (C)	Prednisolone liposomes (LPP)	Triamcinolone liposomes (LTAP)	CY5.5 liposomes	FITC liposomes
Size (nm)	120 ± 5	110 ± 6	110 ± 2	114 ± 2	132 ± 2
PDI	0.014	0.040	0.100	0.060	0.010
Zeta potential (meV)	−0.6	+4.3	+5.5	+0.5	−0.3
Drug concentration	—	5 mg/ml	5 mg/ml	—	—
Encapsulation Efficiency (EE%)	—	10%	10%	—	—

### Preimmunization

A subcutaneous injection of *Mycobacterium tuberculosis* H37Ra antigen (10 mg; Difco, Detroit, MI) suspended in mineral oil (500 μL) was given as preimmunization^32^. One week later, a second injection of the same amount of subcutaneous antigen was given at a separate site. Successful preimmunization was confirmed after one week by the presence of a visible skin nodule at the injection site.

### Induction of experimental uveitis

Experimental uveitis was induced by unilateral intravitreal injection on Day 0 in preimmunized rabbits (7 days after the second preimmunization). The rabbits were anesthetized with intraperitoneal injections of ketamine hydrochloride (35–50 mg/kg) and Xylazil (5–10 mg/kg). Following topical anaesthesia with Amethocaine 1%, the right eye of each rabbit was disinfected with 5% povidone iodine. An intravitreal injection of *Mycobacterium tuberculosis* H37Ra antigen suspended in sterile saline (50 μg; 1 μg/μL) using a Hamilton syringe with a 31-gauge needle was given through the superotemporal sclera, 1.5 mm from the limbus. One drop of Tobramycin was instilled at the end of the procedure. To simulate a recurrence of uveitis, we induced experimental uveitis again on Day 8, following the procedure as described above. The eyes were clinically monitored for 30 days and graded for ocular inflammation by 2 masked investigators.

### Kinetics profile and localization of liposomes

Fluorescent-labelled liposomes were injected subconjunctivally in 4 rabbits to investigate their ocular distribution. Two rabbits received a subconjunctival injection of liposomes labelled with Cyanine 5.5 and were sacrificed 24 h later. Eyes were frozen and sliced for immunostaining and confocal imaging (Nikon center Singapore). Two rabbits were injected with liposomes labelled with FITC to observe the kinetics at the subconjunctival injection site, the cornea and the aqueous humor with a fluorotron imaging. The Fluorotron Master (Fluorophotometry equipment) is approved for human use and this version only differs slightly from the human version in its external features that make it appropriate for positioning to animal eyes. Briefly, 200 μl of FITC labelled liposome solution were injected subconjunctivally into both eyes. Concentration measurements were undertaken with the Fluorotron at baseline, 15 min, 60 min, 48 hours and weeks 1, 2, 3 and 4 post injection for the cornea and aqueous humor sites. Two extra time points (4 h and 24 hours) were added for the subconjunctival injection site.

### Intervention

Rabbits were randomized into 5 groups, 3 days after uveitis induction: a single dose of 0.1 ml subconjunctival LTAP (4 mg/ml) (n = 6), a single dose of 0.1 ml subconjunctival LPP (4 mg/ml) (n = 5), a single dose of 0.1 ml of subconjunctival prednisolone phosphate (FPP) (4 mg/ml), topical Predforte1% Q3H for 2 weeks (ED) (n = 5) or controls (C) (n = 5). Prior to injection, rabbits were anesthetized with intraperitoneal injections of ketamine hydrochloride (35–50 mg/kg) and Xylazil (5–10 mg/kg). Following topical anaesthesia with Amethocaine 1%, the right eye of each rabbit was disinfected with 5% povidone iodine. A Hamilton syringe with a 31-gauge needle was used to deliver subconjunctival injections. Topical Tobramycin was administered 4 times a day for 5 days after the subconjunctival injection.

### Ocular examination

Ocular examination was performed by 2 masked independent investigators (CW, SR). Slit-lamp biomicroscopy, measurement of intraocular pressure with the Tonopen, photography of the anterior segment and dilated fundal examination with binocular indirect ophthalmoscopy using a 20D lens were performed prior to uveitis induction and at 8 defined time points thereafter (Days 0, 1, 3, 4, 8, 9, 11, 16, 24 and 31). Severity of uveitis was scored by evaluating anterior chamber cells/flares, vitreous haze, and iris vessels. These clinical scoring systems had been described in previous literature^33,34^. The combined anterior segment inflammation score was defined as the sum of the scores for iris vessels, anterior chamber cells and anterior chamber flare. The presence of cataract was determined on slit lamp biomicroscopy on day 31 and graded based on the LOCS scale.

### Enucleation, euthanasia and pathology procedures

All rabbits were euthanized at the end of the study period of 30 days. Euthanasia was carried out with intraperitoneal pentobarbitone (60–150 mg/kg) followed by enucleation of the operated eyes.

### Histopathology and immunohistochemistry

Eye were embedded in paraffin or directly frozen (eyes injected with Cy5.5 labelled liposomes). For paraffin embedding, the enucleated rabbit eye was fixed in 10% neutral buffered formalin solution (Leica Surgipath, Leica Biosystems Richmond, Inc.) for 24 hours. The whole rabbit eye was then dissected prior to dehydration in increasing concentrations of ethanol, clearance in xylene, and embedding in paraffin (Leica-Surgipath, Leica Biosystems Richmond, Inc.). Five-micron sections were cut with a rotary microtome (RM2255, Leica Biosystems Nussloch GmbH, Germany) and collected on POLYSINE^TM^ microscope glass slides (Gerhard Menzel, Thermo Fisher Scientific, Newington, CT). The sections were dried in an oven of 37 °C for at least 24 hours. To prepare the sections for histopathological and immunohistochemical examination, the sections were heated on a 60 °C heat plate, deparaffinized in xylene and rehydrated in decreasing concentration of ethanol.

For directly frozen eyes, the whole rabbit eye was embedded in Optimal Cutting Temperature (OCT) compound at −20 °C for 1 hour. Six-micron sections were cut with a cryostat (HM550, Thermo Fisher Scientific Microm International GmbH, Germany) and collected on POLYSINE^TM^ microscope glass slides (Gerhard Menzel, Thermo Fisher Scientific, Newington, CT). Sections were air dried at room temperature (RT) for 1 hour.

A standard procedure for Hematoxylin and Eosin (H&E) was performed. A light microscope (Axioplan 2; Carl Zeiss Meditec GmbH, Oberkochen, Germany) was used to examine the slides and images were captured. In parallel, immunofluorescence staining was performed. For paraffin, heat-induced antigen retrieval was performed by incubating sections in sodium citrate buffer (10 mM Sodium citrate, 0.05% Tween 20, pH 6.0) for 20 minutes at 95–100 °C. The sections were then cooled down in sodium citrate buffer for 20 minutes in RT and washed three times for 5 minutes each with 1x phosphate buffered solution (PBS). For frozen samples, the sections were fixed in 4% paraformaldehyde (PFA) in 1x PBS for 10 minutes and washed three times for 5 minutes each with 1x PBS.

Non-specific sites were blocked with blocking solution of 5% bovine serum albumin (BSA) in 0.1% Triton X-100 and 1X PBS for 1 hour at room temperature in a humidified chamber. The slides were then rinsed briefly with 1x PBS. A specific primary antibody as shown in Supplementary Table S1 was applied and incubated overnight at 4 °C in a humidified chamber prepared in blocking solution. After washing twice with 1x PBS and once with 1x PBS with 0.1% tween for 10 minutes each, Alexa Fluro® 488/594 – conjugated fluorescein-labelled goat anti-rabbit IgG secondary antibody (Invitrogen- Molecular Probes, Eugene, OR) was applied at a concentration of 1:1000 in blocking solution and incubated for 90 minutes at RT. The slides were then washed twice with 1x PBS and once with 1x PBS with 0.1% tween for 5 minutes each, the slides were mounted on the slides with Prolong Diamond Anti-fade DAPI5 Mounting Media (Invitrogen- Molecular Probes, Eugene, OR) to visualize cell nuclei. For negative controls, primary antibody was omitted.

A confocal microscope system (Nikon A1R + si Confocal Microscope) was used to capture high-resolution images. Experiments were repeated in duplicates for four antibodies.

### Statistical analysis

The main outcome measure was the combined clinical scores, defined as the sum of the following scores: (1) iris vessels, (2) anterior chamber cells and (3) anterior chamber flare. Secondary outcome measures were mean intraocular pressure and proportion of eyes with cataract. Statistical analysis was performed using the Statistical Package for Social Sciences (SPSS) version 20 program. Ordinal variables were described with means and analyzed using Mann Whitney U test for independent samples. Proportions were analyzed with the chi square test All p-values are 2 sided with appropriate significance of p < 0.05.

### Data availability statement

The datasets generated during and/or analysed during the current study are available from the corresponding author on reasonable request.

## Results

### Inflammatory scores

**Figure 1:**
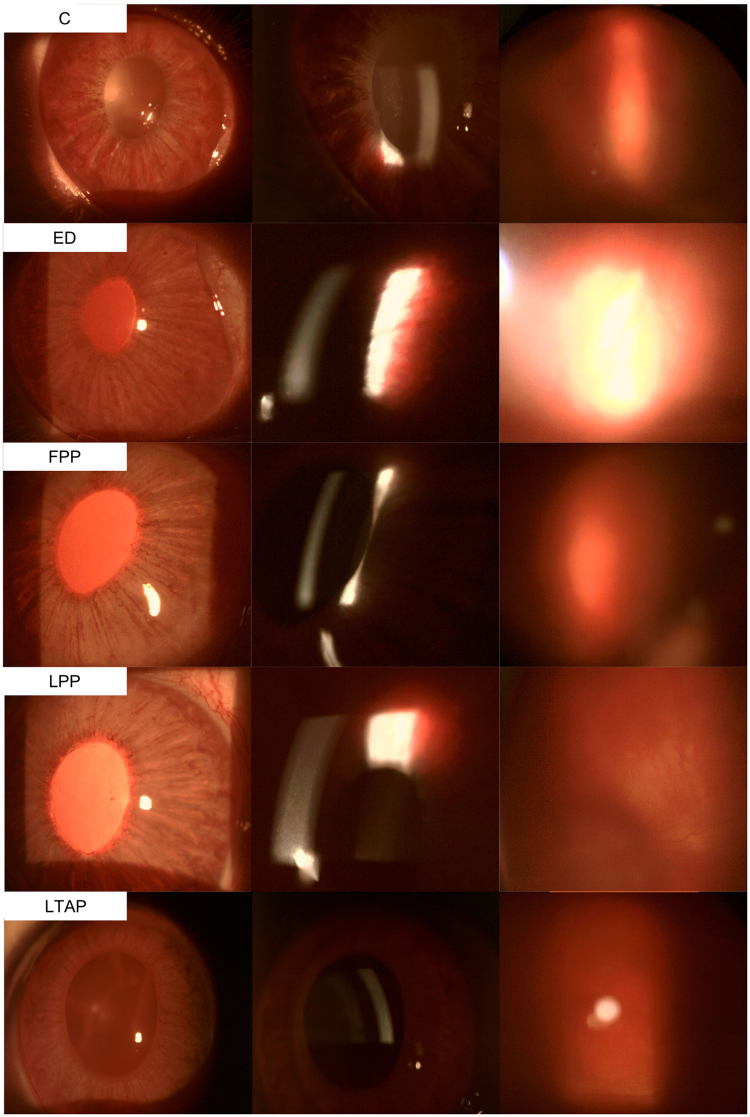
Slit lamp (left and middle rows) and fundus photos (right row) on Day 11. Control eye (top row) had greater iris congestion (top left), anterior chamber cells and flare (top mid) than the eye treated with liposomal PP. Vitreous haze was also worse in the control eye (top right vs 4th row right). Abbreviations: control PBS (C), free prednisolone phosphate (FPP), liposomal prednisolone phosphate (LPP), liposomal triamcinolone phosphate (LTAP), Eye drops treatment (ED).

**Figure 2:**
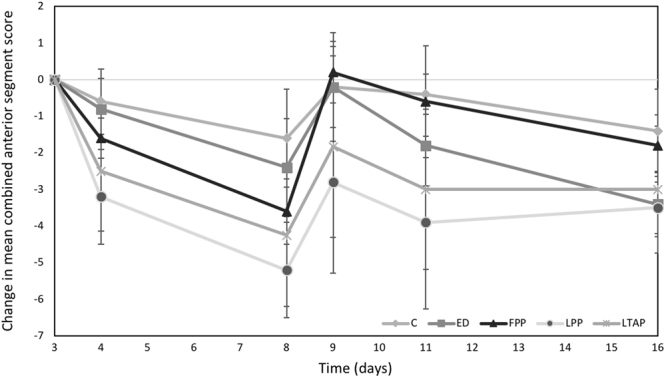
Mean change in combined anterior segment inflammatory scores, (normalization to maximum inflammation on day 3). Treatment started on day 3 with one subconjunctival injection of liposomal prednisolone phosphate (LPP), control PBS (C), free prednisolone phosphate (FPP) or liposomal triamcinolone phosphate (LTAP). Eye drops treatment (ED) started on day 3 with Q3H/4 drops per day until day 16. Recurrence of inflammation was simulated on Day 8 with a repeat challenge of TB antigen.

Table 2 shows the mean combined anterior segment inflammatory scores. One day after subconjunctival injection, the combined anterior segment inflammatory score was significantly lower in the liposomal PP group than in the controls (5.4 ± 1.5 vs 8.4 ± 1.7, p = 0.049), and was also significantly lower than in the eyedrops group (p = 0.033) This difference persisted for 5 days after initial intervention, with both liposomal groups (2.6 ± 2.1, p = 0.019 and 3.3 ± 2.5, p = 0.024 in the liposomal PP and liposomal TA groups respectively) demonstrating significantly lower combined anterior segment inflammatory scores than controls (7.2 ± 2.2). Liposomal PP achieved greater attenuation of rebound inflammation than controls on day 11, 3 days after a rechallenge with intravitreal TB antigen (4.7 ± 2.6 vs 8.5 ± 1.3, p = 0.041). In comparison, while subconjunctival free PP was able to suppress inflammation significantly on day 8 (3.2 ± 0.4), rebound inflammation was observed on day 11 (7.0 ± 2.3). A single dose of subconjunctival liposomal PP or TA delivered sustained anti-inflammatory for 2 weeks post treatment, similar to daily Pred forte eyedrops instilled 4 times a day for 2 weeks (5.0 ± 2.8 and 5.0 ± 1.0 for liposomal PP and TA respectively, vs 4.6 ± 1.3 for eyedrops, p > 0.05). Slit lamp and fundus photographs of all treatment groups are shown in Fig. 1. The control eye showed greater iris congestion, anterior chamber cells and flare and vitreous haze compared to the eye treated with liposomal PP, 1 week after initiation of treatment (Day 11). Figure 2 shows the mean change in combined anterior segment inflammatory scores relative to the maximum inflammation on day 3. Decreases in combined anterior segment inflammatory scores relative to the score on day 3 were greatest in both liposomal groups one day and 5 days after treatment. In addition, there was greatest attenuation of rebound inflammation after antigen re-challenge again in both liposomal groups. On day 16, both liposomal groups achieved a similar decrease in mean inflammatory scores compared to the eyedrop group.

**Table 2:** Mean combined anterior segment inflammatory scores.

Day	Combined anterior segment inflammatory score					
	Liposomal PP (n = 5)	Liposomal TA (n = 6)	Free PP (n = 5)	Pred Forte 1% eyedrops (n = 5)	Controls (n = 5)	^†^P
0	**1st intravitreal induction**					
1	9.4 ± 0.5	9.7 ± 0.5	8.6 ± 0.5	9.0 ± 1.0	9.6 ± 0.5	0.080
	9.4 ± 0.5	9.7 ± 0.5	8.0 ± 0.7	9.0 ± 1.4	9.0 ± 1.4	0.350
3	**Intervention**					
4	5.4 ± 1.5*	6.5 ± 1.9	6.0 ± 0.7	8.0 ± 1.4*	8.4 ± 1.7*	0.020
8	2.6 ± 2.1**	3.3 ± 2.5**	3.2 ± 0.4**	6.0 ± 0.7	7.2 ± 2.2**	0.002
8	**2** ^**nd**^ **intravitreal induction**					
9	5.8 ± 2.7	7.0 ± 2.4	8.0 ± 1.2	8.8 ± 1.3	8.5 ± 2.4	0.130
11	4.7 ± 2.6***	5.5 ± 2.3	7.0 ± 2.3	6.4 ± 0.9	8.5 ± 1.3***	0.041
16	5.0 ± 2.8	5.0 ± 1.0	5.4 ± 1.3	4.6 ± 1.3	7.6 ± 1.9	0.080
24	1.4 ± 1.5	2.2 ± 1.7	2.8 ± 0.4	1.2 ± 1.6	4.0 ± 2.2	0.080
31	0.8 ± 1.8	2.0 ± 2.5	2.2 ± 1.3	1.8 ± 1.9	3.2 ± 1.8	0.440

^†^P values from one-way ANOVA, comparing mean combined anterior segment inflammatory scores between groups.

^*^Pairwise comparison between liposomal PP with controls, p = 0.049 and with eyedrops, p = 0.033. ^**^Pairwise comparison between liposomal PP with controls, p = 0.007, pairwise comparison between liposomal TA with controls, p = 0.019 and pairwise comparison between free PP with controls (p = 0.024).

^***^Pairwise comparison between liposomal PP with controls, p = 0.041.

### Cataract formation

**Figure 3:**
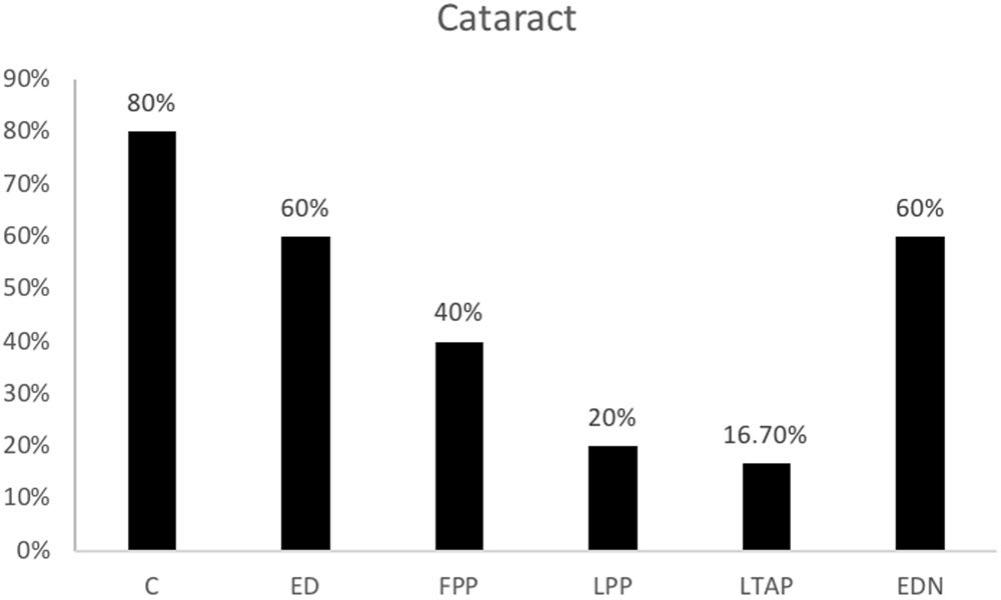
Cataract formation by treatment group. Abbreviations: control PBS (C), free prednisolone phosphate (FPP), liposomal prednisolone phosphate (LPP), liposomal triamcinolone phosphate (LTAP), Eye drops treatment (ED), Eye drops treatment in non-inflamed eye (EDN). Treatment started on day 3 with one subconjunctival injection or eye drops 4 times a day until day 16.

Overall, posterior subcapsular cataracts developed in 11 rabbits. No other subtype of cataract was observed. There was no significant difference in the rate of cataract formation between treatment groups (p = 0.185) but there was a trend towards higher rates in controls, eyedrops and subconjunctival free prednisolone phosphate groups (Fig. 3). No cataracts were seen in fellow eyes administered with prednisolone acetate 1% eyedrops.

### Intraocular pressure

**Figure 4:**
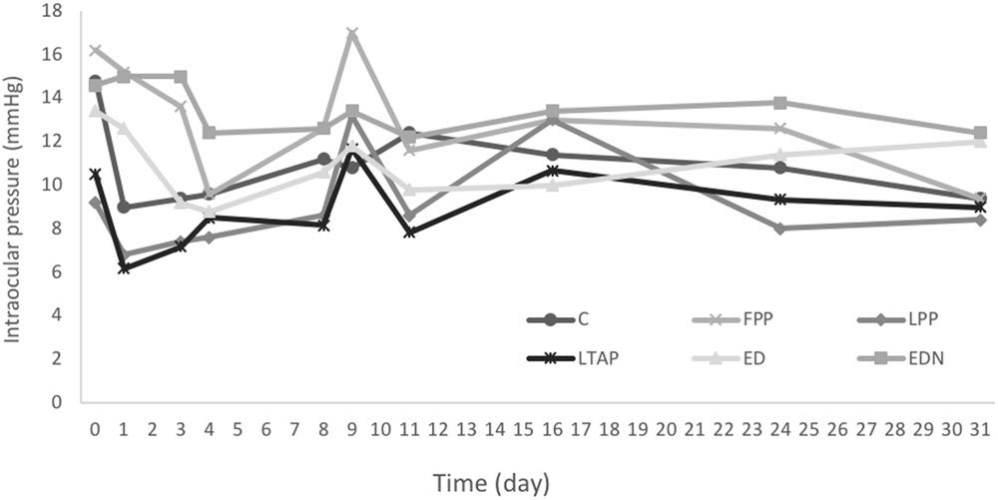
Intra Ocular Pressure (IOP) in each treatment group over time. Control PBS (C), free prednisolone phosphate (FPP), liposomal prednisolone phosphate (LPP), liposomal triamcinolone phosphate (LTAP), Eye drops treatment (ED), Eye drops treatment in non-inflamed eye (EDL). Treatment started on day 3 with a single subconjunctival injection or eye drops 4 times a day until day 16. Recurrence of inflammation was simulated on Day 8 with a repeat challenge of TB antigen.

There were no significant differences in IOP between the treatment groups (Fig. 4) at any time point. A non-significant spike in IOP was observed on day 9, a day after antigen-rechallenge, in all groups except the control group and in the non-inflamed eyes, but IOP remained within normal limits in all eyes. Importantly, none of the rabbits experienced an IOP > 21 at any point during the experiment.

### Histology and immunohistochemical staining

**Figure 5:**
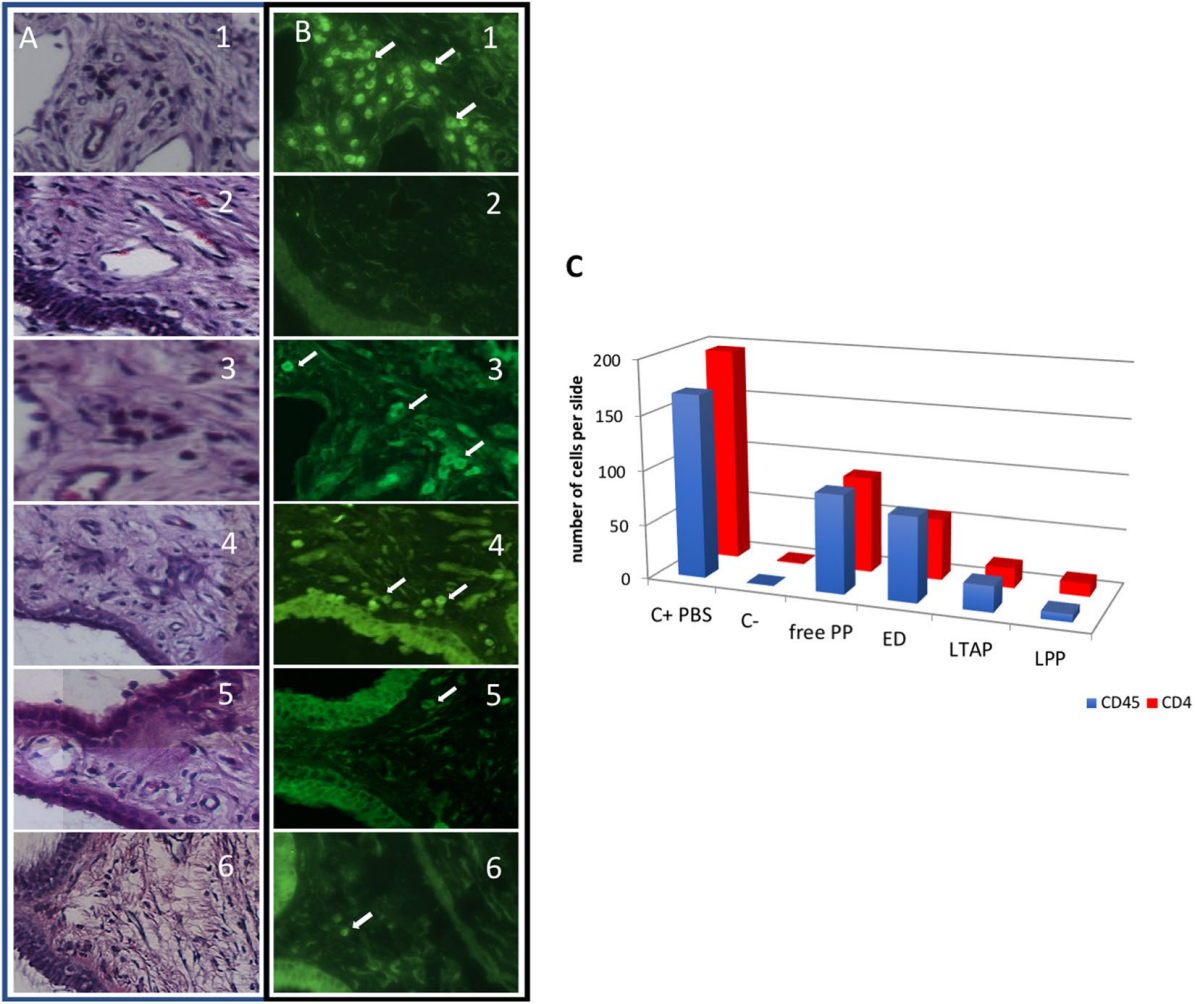
Inflammatory response to treatment, day 30. (**A**) HE staining of each treatment, (**B**) immunostaining with anti-CD45. PBS (1), healthy eye (2), free prednisolone, FPP (3), prednisolone eye drops, ED (4), liposomal triamcinolone phosphate, LTAP (5), liposomal prednisolone phosphate, LPP (6). Inflammatory cells in treated eye (white arrow). (**C**) Mean number of CD45 and CD4 cells present in the ciliary body per slide after treatment on day 30 for each treatment. Control PBS (C+ PBS), normal fellow eye (C−), eyedrops (ED), free prednisolone phosphate (free PP), liposomal triamcinolone acetonide phosphate (LTAP), liposomal prednisolone phosphate (LPP).

The H&E staining showed normal tissue structure in all groups. However, more cellular infiltration (dark purple) was observed in the control group. With immunohistochemical staining, we confirmed ciliary body inflammation in the control group with presence of leucocytes (CD45, Fig. 5B1) and T lymphocytes (CD4). The number of inflammatory cells between groups correlated with the observed inflammatory score (Fig. 5A–C): less inflammatory cells were seen in the LPP and LTAP groups compared with ED, free PP or controls at 30 days post uveitis induction (Fig. 5C).

### Localisation of liposomes in inflamed areas

**Figure 6:**
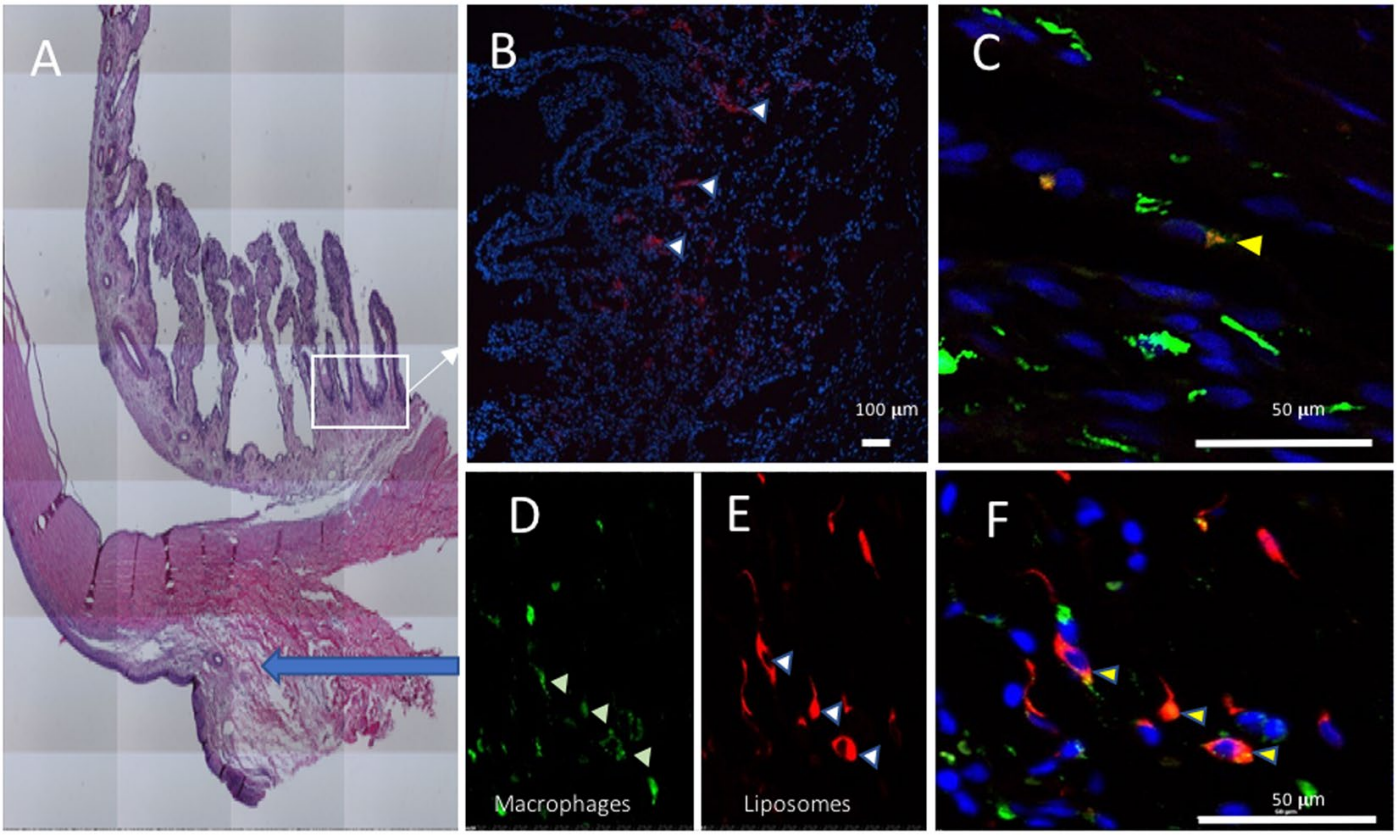
Localization of liposomes in PBS group (Control) on day 4, 24 hours after liposomes CY5.5 injection. (**A**) HE staining on the anterior segment, in paraffin. Blue arrow represents the subconjunctival injection site. White square represents the ciliary body. (**B**,**C**) Confocal imaging focused on the ciliary body (white square), frozen tissue. (**D**,**E**,**F**) Confocal imaging focused on subconjunctival injection site (blue arrow) (**D**) staining of macrophages (green), (**E**) liposomes (red), (**F**) overlay. Liposomes are represented in red (white arrows), nucleus in blue (DAPI), macrophages in green (Alexa 594), yellow arrow shows the co-localisation of macrophages and liposomes.

24 hours after injection of CY5.5-liposomes, fluorescence was detected in the ciliary body as well as the subconjunctival injection area. After immunostaining, fluorescence was detected within macrophages (Fig. 6).

### Liposome kinetics

**Figure 7:**
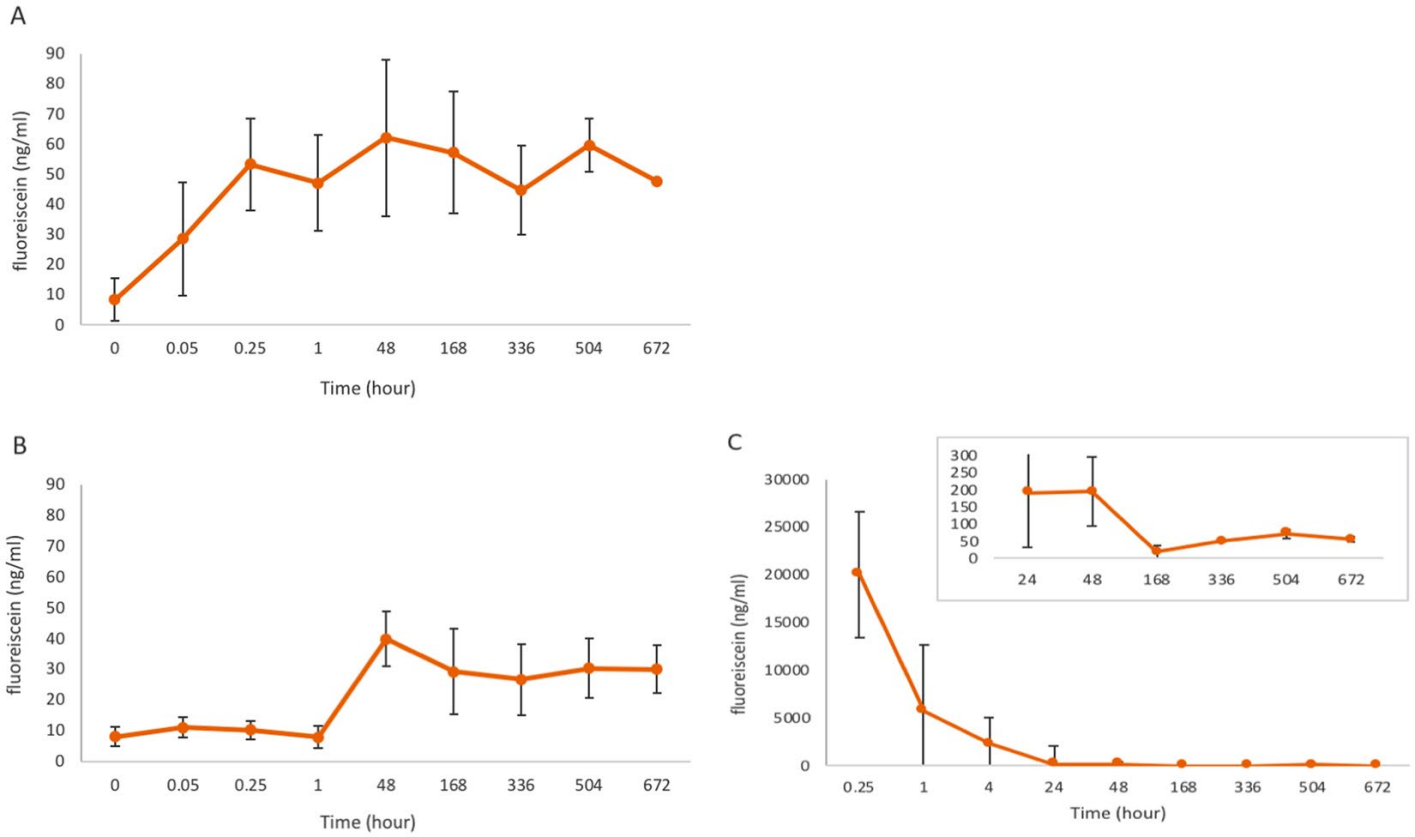
Kinetics of FITC- labelled liposomes after subconjunctival injection. Mean fluorescein concentration (ng/ml) measured over time (hours) with fluorophotometry. (**A**) in the cornea, (**B**) in aqueous, (**C**) in the subconjunctival

FITC labelled liposomes, after injection in the subconjunctival space, showed a fast elimination during the first day followed by slow elimination persisting over the entire duration of the experiment from the subconjunctival area. A lower quantity of FITC-labelled liposomes was detected in the cornea and this was maintained over 4 weeks. In the aqueous humor, an equivalent quantity was measured and maintained over time, but FITC was detected only after 24 hours post subconjunctival injection (Fig. 7).

## Discussion

In this study, we demonstrated that a single dose of liposomal steroid, injected subconjunctivally, was able to provide sustained anti-inflammatory action comparable to 2 weeks of eyedrop therapy with prednisolone acetate 1%. Importantly, we found that liposomal prednisolone phosphate was able to suppress the initial inflammation better than eyedrops (p = 0.033). This is likely the result of a more rapid build-up of therapeutic levels within the eye via the subconjunctival route compared to topical administration. The subconjunctival route may also reduce ocular irritation associated with benzalkonium chloride, a preservative commonly found in topical eyedrops. Further, we observed that liposomal steroid was able to sustain anti-inflammatory action and attenuate an antigen rechallenge, an effect that was not achieved by subconjunctival injection or topical application of free steroid. Subconjunctival injections can be given relatively easily and painlessly in the outpatient setting under topical anaesthesia. There is little risk of globe injury compared to peribulbar injections. Moreover, as there is no intraocular penetration, subconjunctival administration does not entail the risk of endophthalmitis that is associated with intravitreal or intracameral injections.

A myriad of drug delivery approaches has previously been studied for the delivery of corticosteroids to treat anterior uveitis. These include cubosomes^35^, micellar systems, implant^12^, a variety of nanoparticles, microemulsions, and iontophoresis. At present, only iontophoresis has been evaluated in phase III trials for the treatment of anterior uveitis, demonstrating non-inferiority when compared to intensive topical eyedrop therapy.

Liposomal steroids, whether local or systemic, have not been previously assessed for the treatment of anterior uveitis. Liposomes are one of the more successful drug delivery platforms for ocular diseases that have made it to human clinical trials, including their application to treat dry eyes^36^, allergic rhinoconjunctivitis^37^ and cytomegalovirus infection of the retina^38^. In earlier studies, Pouvreau *et al*.^22^ and Broekhuyse *et al*.^21^ have observed a significant anti-inflammatory effect on experimental uveitis after depletion of macrophages with dichloromethylene diphosphonate (Cl2MDP)-containing liposomes. These results suggest that liposomes may have the advantage of preferential uptake by macrophages, the major cell type involved in anterior uveitis, as demonstrated in our study with the observation of co-localisation of liposomes and macrophages.

An added advantage of the subconjunctival administration route may be the avoidance of ocular side effects. While steroids are crucial for the adequate suppression of inflammation-related, they can cause sight-threatening complications such as raised intraocular pressure and cataracts. Previous studies have shown that 30% of all steroid-treated eyes may experience elevation of intraocular pressure after prolonged topical steroid treatment. This side effect is related to a direct effect of steroids on the extracellular matrix proteins in the trabecular meshwork and the inhibition of phagocytosis by trabecular meshwork cells, both of which cause a reduction in aqueous outflow^39,40^. In our study, none of the eyes treated with liposomal steroids developed raised intraocular pressure throughout the duration of the study. Preferential uptake of liposomes by macrophages may reduce the total dose required for sufficient efficacy. In addition, encapsulation of steroids within liposomes may ameliorate some of the deleterious side effects when the trabecular meshwork is exposed to steroids. With regards to cataract formation, most eyes developed some degree of posterior subcapsular cataract in this study as a result of the inflammatory process, with fewer eyes experiencing cataract formation in the liposomal steroid groups. This can be attributed to the better control of inflammation in these eyes. None of the fellow eyes treated with liposomal steroid developed cataracts. Again, this may be attributed to the encapsulation of the steroid thus avoiding this adverse effect, but it may also result from the overall lowering of the dose of steroid via the single subconjunctival injection.

The tear film, cornea and anterior chamber and capillaries of the iris collectively form the ocular barriers to eye drop drug delivery for the treatment of anterior uveitis^41,42^. With subconjunctival injection, drug entry can potentially bypass the aforementioned barriers and, via crossing the sclera, reach the ciliary body, one of the two main target sites in anterior uveitis, the other being the iris. Indeed, liposomes have been reported to be able to cross the sclera and to reach the vitreous in intact form^16,17^. However, quantitatively, the extent to which the trans-scleral route permits drug delivery into the ciliary body is not known, and will most likely depend on the physicochemical properties of the drug delivery system in question. Drug binding to scleral melanin may form yet another barrier to drug delivery via the trans-scleral route. Regarding the mechanism behind the rapid and long lasting effect of both liposomal steroid preparations, we can only speculate about the various possible routes through which intact liposomes and steroid (still entrapped or released) reach the target inflammation areas (in the iris and the ciliary body) It is well established^26,27,30^ that the type of liposomes used, PEGylated liposomes, can be taken up by inflammatory macrophages present in inflammatory lesions. Our initial histological and immunochemical staining results confirm that the PEG-liposomes co-localise with macrophages at the injection site and in the ciliary body. Within these macrophages, the steroid-containing PEG-liposomes are degraded intracellularly. This intracellular degradation process occurring in the lysosomes liberates the entrapped steroid from the liposomal structures and released drug molecules are then able to diffuse throughout the cellular interior, and are possibly even released by the macrophages into the environment^43,44^. The released drug molecules act intracellularly to reduce the pro-inflammatory activity of these macrophages^26–28,31^. In addition to this mechanistic option, the administered PEG-liposomes could act as a depot slowly releasing its steroid content extracellularly. The exact sites of extracellular drug release are not known at present, but it has been previously described that, after subconjunctival injection of nanoparticles, released drugs are able to reach the ciliary body via the conjunctiva, tears and aqueous humour and also through the sclera and vitreous humor^9^. Furthermore, the fluorotron results showed persistence of fluorescein (FITC-labelled liposomes) in the subconjunctival injection area over the entire duration of the experiment, indicating a sustained presence of liposomal nanoparticles at the injection site. This supports the notion that liposomes could act as a drug reservoir slowly releasing the drug at the injection site. We also detected a low but steadily maintained fluorescein quantity in the aqueous humour and in the cornea throughout the duration of the experiment.

There are limitations to our study. The study is not powered to study adverse effects. In addition, the follow up duration may be too short to identify the development of long term complications such as cataracts and raised IOP. However, a single subconjunctival injection of steroid is unlikely to induce cataracts, a complication seen with chronic topical steroid use or with intravitreal administration. Similarly, steroid induced IOP elevation is usually observed after a substantial period (several weeks to months) of topical steroid use^39^, and is due to alterations in trabecular outflow resistance as discussed above^40^. This phenomenon may not be inducible in our animal model: a previous study failed to incite IOP elevation in rabbits with topical steroids applied 4 times a day for 1 month^40^. We propose that encapsulation of steroids in liposomes will reduce the total dose needed to achieve efficacy, hence minimizing the exposure of the trabecular meshwork to the effects of steroids, and further reduce the likelihood of steroid-induced IOP elevation.

In conclusion, our study in a rabbit anterior uveitis model demonstrates that a single subconjunctival injection of liposomal steroids is capable of inducing effective and sustained anti-inflammatory action, and to attenuate the effects of a simulated recurrence of uveitis. Subconjunctival injections can be administered in the clinical setting safely with relative ease and without the need for sophisticated equipment. Our results suggest that a single subconjunctival injection of liposomal steroid represents an attractive option for the treatment of anterior uveitis and since these formulations are already under clinical investigation in other indications and administration routes, rapid translation of our preclinical results to a first-in-human trial may be possible.

## Electronic supplementary material

Antibodies used for histological examination.
