# Supplementary material for: Evaluation of subconjunctival liposomal steroids for the treatment of experimental uveitis
Source: Sci Rep. 2018 Apr 26;8:6604. doi: 10.1038/s41598-018-24545-2 (PMC5919899; doi:10.1038/s41598-018-24545-2)
Supplement: Supplementary file 1 — Antibodies used for histological examination. [file 41598_2018_24545_MOESM1_ESM.doc]

**Evaluation of subconjunctival liposomal steroids for the treatment of experimental uveitis**

**Authors:** Chee Wai Wong, MMed(Ophth)^1,2†^, Bertrand Czarny, PhD^3,4,5 †^, Josbert M. Metselaar, PhD^6^, Candice Ho^2^, Ng Si Rui, MBBS ^1,2^, Amutha Barathi Veluchamy, PhD^2^, Gert Storm, PhD^3,6*^, Tina T. Wong, PhD^1,2*^.

**Affiliations:**

1. Singapore National Eye Centre (SNEC), 11 Third Hospital Avenue, Singapore 168751

2. Singapore Eye Research Institute, 11 Third Hospital Avenue, Singapore 168751

3. Dept. Pharmaceutics, Utrecht Institute for Pharmaceutical Sciences (UIPS), Utrecht University, PO Box 80082, 3508 TB Utrecht, The Netherlands.

4. School of Materials Science and Engineering (MSE), Nanyang Technological University, 11 Faculty Avenue, Singapore 639977

5. Lee Kong Chian school of medicine (LKCmedicine), Nanyang Technological University, 11 Mandalay Road, Singapore 308232

6. Department of Experimental Molecular Imaging, University Clinic and Helmholtz Institute for Biomedical Engineering, RWTH Aachen University, Aachen 52074, Germany

† These authors contributed equally to this work.

* Corresponding author: Email: [tina.wong.t.l@snec.com.sg](mailto:tina.wong.t.l@snec.com.sg); Email: [G.Storm@uu.nl](mailto:G.Storm@uu.nl)

Address: Singapore National Eye Centre, 11 Third Hospital Avenue, Singapore 168751 Telephone: (65) 63227477

Fax: (65) 62252568

Supplementary table S1: Antibodies used for histological examination.

| **Antibody** | **Catalog No.** | **Company** | **Concentration** |
| --- | --- | --- | --- |
| Macrophage clone RAM11 | M0633 | Dako | 1:50 |
| CD4 | 553043 | BD Pharmigen (Franklin Lakes, NJ) | 1:50 |
| CD45 | sc-70690 | Santa Cruz (Santa Cruz Biotechnology, Santa Cruz, CA) | 1:50 |
| Alexa Fluor 488 goat anti−mouse IgG (H+L) | A11001 | Invitrogen. Life Technologies (Invitrogen, Eugene, OR) | 1:1000 |
| Alexa Fluor 488 goat anti−rat IgG (H+L) | A11006 | Invitrogen. Life Technologies (Invitrogen, Eugene, OR) | 1:1000 |
| Alexa Fluor 594 goat anti−mouse IgG (H+L) | A11032 | Invitrogen. Life Technologies | 1:1000 |
| Alexa Fluor 594 goat anti−rat IgG (H+L) | A11007 | Invitrogen. Life Technologies | 1:1000 |
